# Recognizing scientific excellence in the biology of cell adhesion

**DOI:** 10.1186/1478-811X-3-7

**Published:** 2005-04-18

**Authors:** Kishore K Wary

**Affiliations:** 1 Center for Extracellular Matrix Biology, Institute of Biosciences and Technology, Texas A & M University System-Health Science Center, Texas Medical Center, 2121 W. Holcombe Blvd. Houston, TX-77030, USA

## Abstract

The prestigious 2005 Japan Prize for Cell Biology has been awarded to Dr. Masatoshi Takeichi, Director of RIKEN Developmental Biology, Kobe, Japan, and Dr. Erkki Ruoslahti, Distinguished Professor, The Burnham Institute, La Jolla, USA for their "Fundamental contribution in elucidating the molecular mechanisms of cell adhesion". The award is scheduled to be presented to the scientists in ceremonies in Tokyo on April 20, 2005 as part of a week-long celebration of "Japan Prize Week".

## What is cell adhesion?

Well, why does our skin look so smooth on the surface? How do skin cells adhere to each other and the underlying connective tissue to resist wound and bruise? How do two 'unlike' or 'like' cells live side-by-side? How are muscles and tendons glued to the bones? How do endothelial and epithelial cells are separated from each other? What mechanisms divide astrocytes, neurons, and the endothelial cells that make up the neurovascular unit? The answer is "cell adhesion", which is because of the characteristic properties of proteins and molecules that act like 'glue' or 'sticky molecules'. If cells or tissues do not hold each other, like in blistering skin in which something as gentle as a human touch can cause the skin to blister and peel away, inviting fatal infection and wound that may never heal. Suffice to say, the chances of survival will be somewhat diminished.

## What are cell adhesion molecules?

In the late 1970's two ideas were put forward. First, the chemoaffinity hypothesis proposed that cell-cell contacts are mediated by unique set of cell adhesion molecules presented by adjacent cells. Second, adhesion molecules are limited, but their affinity could switch from low to high and *vice versa*. Soon afterwards, several important cell adhesion molecules were discovered and described including the cadherins, neuronal cell adhesion molecules (NCAM), extracellular matrix (ECM) molecules, proteoglycans, the immunoglobulin cell adhesion molecules, junctional adhesion molecules (JAMs), connexins, and selectins. Those ideas are very much alive and many cell adhesion molecules discovered recently are being tested with stringent criteria with better technologies today.

## How do these molecules promote cell adhesion?

**Figure 1:**
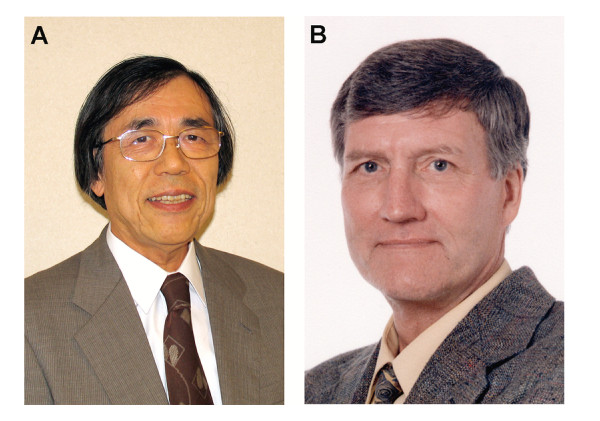
**A **Dr. Masatoshi Takeichi (left), and **(B) **Dr. Erkki Ruoslahti (right). Image (A) is provided by Dr. Takeichi, and (B) obtained from public domain.

**Figure 2:**
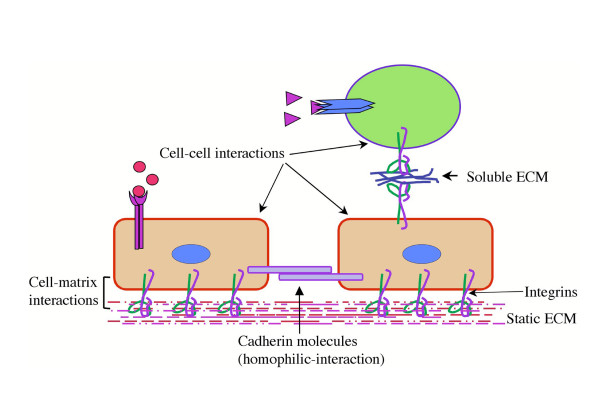
**Schematics of cell adhesion mediated by cadherin and by extracellular matrix (ECM) proteins. **Cadherin molecule connects adjacent cells by homophilic interactions in a metal ion dependent manner. Integrin cell adhesion receptors can interact with both static as well as soluble ECM ligands. In addition, integrins can also bind cell-associated ligands (not shown).

**Figure 3:**
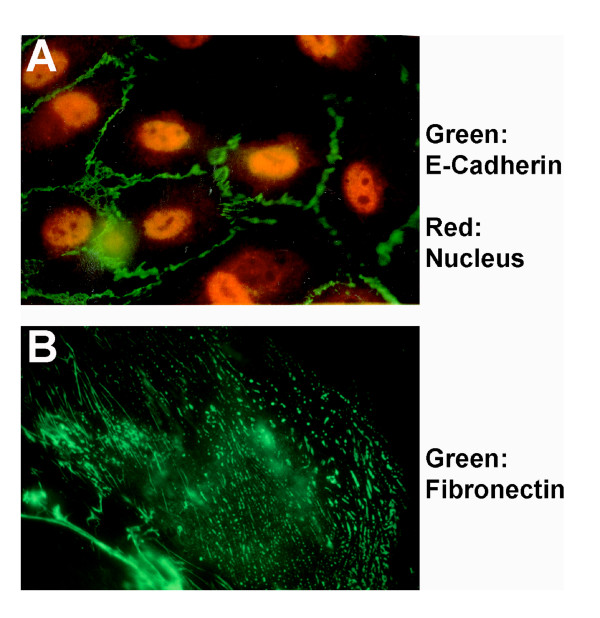
**(A) Cell-cell adhesion- **Epithelial cells were stained with anti-E-cadherin monoclonal antibody and detected by fluorescent dye microcopy. Green fluorescent represents E-cadherin molecules connecting cells. Red color represents nucleus. **(B) Cell-matrix adhesion**- Adherent endothelial cell was stained with anti-fibronectin monoclonal antibody and detected by fluorescent microscopy. Images are not in scale, magnification, 200×

There may not be a unifying answer to that question. In one of the landmark articles, Dr. Masatoshi Takeichi [Fig. 1A] described calcium-dependent and -independent mechanisms of cell adhesion [1]. Cell-aggregation assays of disaggregated tissue and cells provided indication that the cadherins promote 'homophilic' interactions, a process that requires presence of Calcium metal ions [1-3]. Cadherins are transmembrane proteins containing an extracellular, a transmembrane, and a cytoplasmic segment. The extracellular domains of cadherins mediate Calcium-dependent intercellular adhesion by homophilic interactions. The binding properties and specificities of the adhesive interactions are located in the N-terminal segment of the molecules. A total of 17 classical cadherins have been described in the literature. Cadherin superfamily is made of 85 members. The classical cadherins are mainly involved in the cell adhesion. The roles of the other members of cadherin superfamily remain to be elucidated. Cell adhesions mediated by cadherins are cell type specific. In epithelial and endothelial cells, cadherins mediate formation of adherens junctions. It is now clear that the intracellular signaling components of cadherin determine the epithelial morphogenesis and tissue architectures [2-5]. The loss of cadherin expression by neoplastic cells is a hallmark of tumor progression [6]. Dr. Erkki Ruoslahti [Fig 1B] provided evidence that most ECM molecules such as fibronectin [7] promote both cell-cell and cell-matrix interaction by interacting with a family of cell adhesion receptor called the integrins [8]. In contrast to static ECM, some of the soluble ECM molecules can serve as a 'bridge' between two like or unlike cells [Fig. 2]. Such interactions are both transient as well as static, for example, at the sites of injury and inflammation, and these interactions could be low or high affinity [9]. The development of specific monoclonal antibodies such as (Ligand-induced binding site, LIBS, and cation- and ligand-induced binding site, CLIBS) as well as fluorescence energy transfer experiments provided further clues to the nature of the molecular interactions of integrin with the ECM molecules [9]. Moreover, molecular genetic analyses have provided evidence that multicellular organisms are dependent on adhesion of cells to each other and the ECM molecules, without which many cells will fail to stick [10]. Accordingly, gene deletion studies in mouse embryos have provided evidence that both cadherin and fibronectin molecules are required for embryonic development. The studies of cultured cells have provided early evidence that both fibronectin and cadherins help organize the cytoskeleton. In short, the prize is all about elucidating the molecular mechanisms as to how cell adhesion works [Fig. 2, 3].

## Cell-aggregation and cell adhesion assays

Cell biologists use proteolytic enzymes such trypsin and Ethylenediaminetetra-acetic acid (EDTA) to detach/disaggregate cells from the culture dishes and to prepare of primary cells from intact tissues. Trypsin is a proteolytic enzyme, while EDTA, a metal ion chelator. When used in right combination, they can disrupt both cell-cell and cell-matrix interactions, that is to say these two substances can disaggregate cells and tissues [1]. Cell-cell and cell-matrix interactions appear to go hand-in-hand [Fig. 2]. Upon attachment adherent cells sense presence of Calcium in the environment, calcium is required for both cell-cell and cell-matrix interactions. Dr. Takeichi described as to how cell-cell interactions between "like" cells and "unlike" cells can be induced by E-cadherin molecules [2,3]. In normal adherent cells such as endothelial (vascular endothelial cadherin, called VE-Cadherin) and epithelial cells (epithelial cadherin, called E-cadherin) cadherins connect two or more cells in a "zipper" like fashion in presence of calcium [1-3,11]. Importantly, calcium prevents the degradation of cadherin and promotes cell adhesion activity [1]. Cadherin may also be important for mediating "contact-inhibition", a property of normal adherent cells. In a nutshell, Dr. Takeichi observed and described calcium-dependent and -independent mechanisms of cell adhesion [1].

Short-term incubation of adherent cells with trypsin can digest most ECM molecules and addition of EDTA helps disrupt interactions of cell adhesion mediated by integrins [8]. Dr. Ruoslahti described purification and characterization of fibronectin from blood plasma, and went onto identify the Arg-Gly-Asp (RGD) tri-peptide cell adhesion motif, that remains as a conceptual breakthrough [7,12]. Structurally, the fibronectin represents a prototypic ECM molecule that displays highly modular structure [13]. Fibronectin possesses repeating structural motifs, classified as fibronectin repeats FN-I, FN-II, and FN-III that are grouped together into functional domains [12,13]. Many cell types secrete fibronectin polypeptide ranging between 220–250 kDa sizes. Functionally, fibronectin plays critical roles in cell adhesion-dependent cellular activities both in development and adult tissue homeostasis, proliferation, and migration [12,13]. Cell adhesion assays provided evidence that cell attachment mediated by specific subset of integrins onto fibronectin can be blocked by RGD tri-peptides. Subsequently, many laboratories around the world have also cloned, characterized and described ECM proteins that also may contain RGD or variant cell binding sites. Detergent solubilized plasma-membrane proteins passed through a column chromatography conjugated to RGD-peptides allowed purification of RGD-binding cell adhesion receptors, which is now known as integrins [14]. For detail, please see the RGD story by E. Ruoslahti [14]. Secreted fibronectin can organize and assemble large protein complex by interacting with many other ECM molecules in the extracellular space such as the fibrinogen and collagens, glycosaminoglycans, proteoglycans, tenascin, fibulin and thrombospondin. Many normal cells have been described as "anchorage-dependent" cells that require attachment factor such as fibronectin, without which cells die of apoptosis [15,16]. Apart from just being a structural support, Fibronectin can trap or sequester growth factors and cytokines, and induce signaling activities [16,17]. In contrast, neoplastic cells that accumulate mutant copies of genetic materials no longer require fibronectin to grow, divide or metastasize, and this phenomena is called "anchorage-independence". Fibronectin also interacts with bacterial adhesion molecules, a pathological process that helps bacteria to colonize and infect host tissues [18].

## Conclusion

In addition to providing structural support, both cadherins and fibronectin molecules are also required for cell polarity, and informing the cells and tissues about their position in time and space, called positional cues [17]. A biological process that allow cells to sense their immediate physical and chemical environment correctly, for example, to help cells sense presence of glucose and insulin, cytokines and growth hormones, signaling molecules and metal ions. However, such regulatory mechanisms could be altered in many pathological states including tumor growth, angiogenesis and metastasis. Complete understanding of the mechanisms of regulation of cell-adhesion molecules and their signaling activities remains an active area of investigation in many disease settings including cardiovascular, cancer, neural networks, damage and repair mechanisms associated with traumatic injury, wound healing, host-pathogen interactions, nanotechnology, tissue engineering, and molecular therapeutics. The Japan Prize 2005 and cash award is slated to be given in presence of the Emperor of Japan in a week-long celebration beginning 20^th ^April, 2005 .

## Authors' contributions

K.K.W. wrote and edited the whole manuscript.
